# Feeding Habits of Leopards and Leopard Cats in the Fragmented Forests Surrounding the Kathmandu Valley

**DOI:** 10.1002/ece3.70927

**Published:** 2025-01-29

**Authors:** Prajwol Manandhar, Keren S. Pereira, Naresh Kusi, Jyoti Joshi, Noam Levin, Hemanta K. Chaudhary, Claudia Wultsch, Sandesh Lamichhane, Suman Bhandari, Laba Guragain, Rajesh M. Rajbhandari, Berndt J. V. Rensburg, Salit Kark, Dibesh Karmacharya

**Affiliations:** ^1^ Center for Molecular Dynamics Nepal Kathmandu Nepal; ^2^ School of the Environment University of Queensland St Lucia Queensland Australia; ^3^ SOLARIS Trail Cameras Brisbane Queensland Australia; ^4^ Himalayan Wolves Project Salenstein Switzerland; ^5^ Department of Forestry and Wildlife Management University of Inland Norway Elverum Norway; ^6^ Department of Geography The Hebrew University of Jerusalem Jerusalem Israel; ^7^ Remote Sensing Research Centre, School of the Environment University of Queensland Brisbane Queensland Australia; ^8^ Bioinformatics and Computational Genomics Laboratory, Hunter College City University of New York New York New York USA; ^9^ Sackler Institute for Comparative Genomics American Museum of Natural History New York New York USA; ^10^ School of Forestry and Natural Resource Management, Institute of Forestry Tribhuvan University Kathmandu Nepal; ^11^ Shivapuri Nagarjun National Park Department of National Parks and Wildlife Conservation Kathmandu Nepal

**Keywords:** DNA metabarcoding, feeding ecology, habitat disturbance, Kathmandu Valley, *Panthera pardus*, *Prionailurus bengalensis*

## Abstract

Large‐scale anthropogenic developments in the metropolitan areas of Nepal and the rural to urban influx of people have exacerbated human–wildlife conflicts across human‐altered landscapes of Nepal. The Kathmandu Valley has experienced large‐scale urbanization and has subsequently witnessed substantial incidents of human–wildlife conflicts given the increasing levels of human encroachment into remnant wildlife habitats. Here, we applied DNA metabarcoding in combination with geospatial analysis to study the feeding ecology of two urban carnivores, the leopard ( *Panthera pardus* ) and the leopard cat ( *Prionailurus bengalensis* ), in the forests surrounding the Kathmandu Valley and to check whether the leopards' predation on domestic animals contributes to human‐leopard conflict in this region and to obtain a baseline data on the dietary habits of the poorly studied leopard cat. We found that leopards were highly dependent on domestic animals in areas dominated by human‐use activities (agricultural and built‐up areas), whereas leopard cats mostly predated on wild rodents. Through our work, we highlight the importance of domestic prey in the diets of urban carnivores like leopards and demonstrate the influence human‐induced habitat disturbance has on the ecology of local wildlife. This study generates critical information which will help to inform conflict mitigation strategies and conservation planning for the two carnivore species, in addition to identifying areas within the region that are susceptible to human–wildlife conflicts.

## Graphical abstract

Large‐scale urbanization and the rural‐to‐urban influx in Nepal have increased human–wildlife conflicts, particularly in the Kathmandu Valley. Using DNA metabarcoding and geospatial analysis, we studied the feeding ecology of leopards and leopard cats, finding that leopards rely heavily on domestic animals in human‐dominated areas, whereas leopard cats primarily prey on wild rodents. This study highlights the impact of human‐induced habitat disturbance on urban carnivores and provides critical information for conflict mitigation and conservation planning.

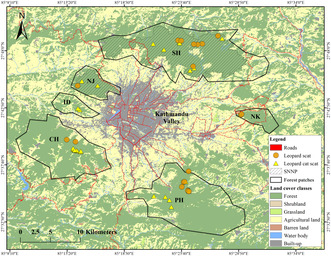

## 1 Introduction

Human–wildlife interactions occur when humans and wildlife encounter one another, often resulting in positive or negative consequences for humans, wildlife, and their resources (Dickman 2010; Nyhus 2016; Soga and Gaston 2020). Negative interactions are commonly labeled as human–wildlife conflict, impacting either or both parties adversely (Conover 2001; Nyhus 2016). From a social science perspective, distinguishing between negative impacts and true conflicts becomes crucial, involving conflicting perceptions about wildlife management (Athreya et al. 2020). Recognizing this, the integration of social science perspectives has gained significance. It not only emphasizes the need to study human–wildlife interactions and their impacts but also addresses conflicting perceptions, providing more effective management recommendations. In this context, we term the conflict between humans and wildlife, encompassing actions adversely affecting either party and perceived threats to human life, property, livestock, and economic security, as human–wildlife conflict. In the developing world, human–wildlife conflict results from the expansion of anthropogenic development into natural habitats and wildlife species' increased adaptability to human‐dominated landscapes (Macdonald and Sillero‐Zubiri 2002; Manfredo 2015; Anand and Radhakrishna 2017). Habitat fragmentation, reducing natural areas and prey populations, increasing habitat edges, and subdividing contiguous patches often increases negative human–wildlife interactions (Laurance 2000; Broadbent et al. 2008; Acharya et al. 2017).

Across much of Asia, leopards ( *Panthera pardus* ) and leopard cats ( *Prionailurus bengalensis* ) exhibit extensive distribution ranges due to their adaptability in habitat selection (Nowell and Jackson 1996; Shehzad et al. 2012; Bashir et al. 2014; Henschel et al. 2015; Pokharel 2015; Laguardia et al. 2017). According to the IUCN Red List, leopards are classified as “Vulnerable” (Stein et al. 2024), whereas leopard cats are listed as “Least concern” (Ghimirey et al. 2023). Leopards, particularly susceptible to conflicts with humans due to their large home ranges and ability to prey on livestock (Macdonald and Sillero‐Zubiri 2002; Athreya et al. 2016; Nyhus 2016), have adapted well to human‐dominated landscapes, even inhabiting major cities like Mumbai and Bengaluru (Athreya et al. 2016; Gubbi, Sharma, and Kumara 2020). These factors contribute significantly to the heightened human–leopard conflict in South Asian countries such as Sri Lanka, India, and Nepal over the past two decades (Fernando 2016; Anand and Radhakrishna 2017; Adhikari et al. 2020). In Nepal, where the human–leopard conflict has intensified, the leopard, an apex carnivore, is the largest felid in the mid‐hills topographic regions (Acharya et al. 2016). The leopard cat, although of lesser concern, is treated as a pest species due to its predation impact on the poultry sector across its distribution range (Kumara and Singh 2007; Jnawali et al. 2011; Rai et al. 2018).

In addition, Nepal's largest metropolitan area, the Kathmandu Valley, has undergone significant land‐use changes over the past three decades due to extensive human migration (Ishtiaque, Shrestha, and Chhetri 2017; Rimal et al. 2017). Most leopard research in Nepal has focused on protected areas (Thapa 2011; Koirala et al. 2012; Maharjan, Shahnawaz, and Shrestha 2017), leaving a gap in understanding the species outside these areas, which constitute over 60% of their habitat and often overlap with human‐use zones (Jnawali et al. 2011; Adhikari et al. 2020; Kandel, Lamichhane, and Subedi 2020). The ecology of the elusive leopard cat is poorly known, especially in human‐modified habitats (Shehzad et al. 2012), complicating the assessment of habitat transformation threats (Mohamed et al. 2013). Anecdotal records alone are insufficient for addressing critical ecological questions.

Diet analysis, particularly through scat analysis, offers vital insights into predation patterns (Klare, Kamler, and Macdonald 2011). Although morphological identification has been the traditional approach, genetic‐based diet analysis or DNA metabarcoding is gaining prominence. The precision of morphological identification can be limited due to observer bias and difficulties in distinguishing closely related species or identifying undigested prey parts (Casper et al. 2007; Mumma et al. 2016; Gosselin, Lonsinger, and Waits 2017; Granquist et al. 2018). This shift may impact research quality, potentially hindering the development of effective management and conflict mitigation strategies in affected regions.

In our study, we focused on assessing leopard diets to understand their potential role in driving human–leopard conflict in the forests surrounding the Kathmandu Valley, aiming to aid the authorities in developing effective conflict mitigation strategies. Additionally, we investigated the foraging ecology of leopard cats to establish a baseline of their diet ecology in this human‐modified area alongside leopards. Employing non‐invasive genetic sampling and next‐generation sequencing, proven successful in similar studies (Shehzad et al. 2012; De Barba et al. 2014; Biffi et al. 2017), we determined carnivore dietary habits. Simultaneously, through geospatial analysis, we characterized land cover types and habitat disturbance in the forests surrounding the Kathmandu Valley. This landscape characterization enables us to pinpoint areas potentially more susceptible to human–leopard encounters. Our comprehensive approach contributes valuable insights for wildlife management, aligning with the broader goal of fostering harmonious coexistence between humans and carnivores in this dynamic landscape.

## 2 Materials and Methods

### 2.1 Study Area

**FIGURE 1:**
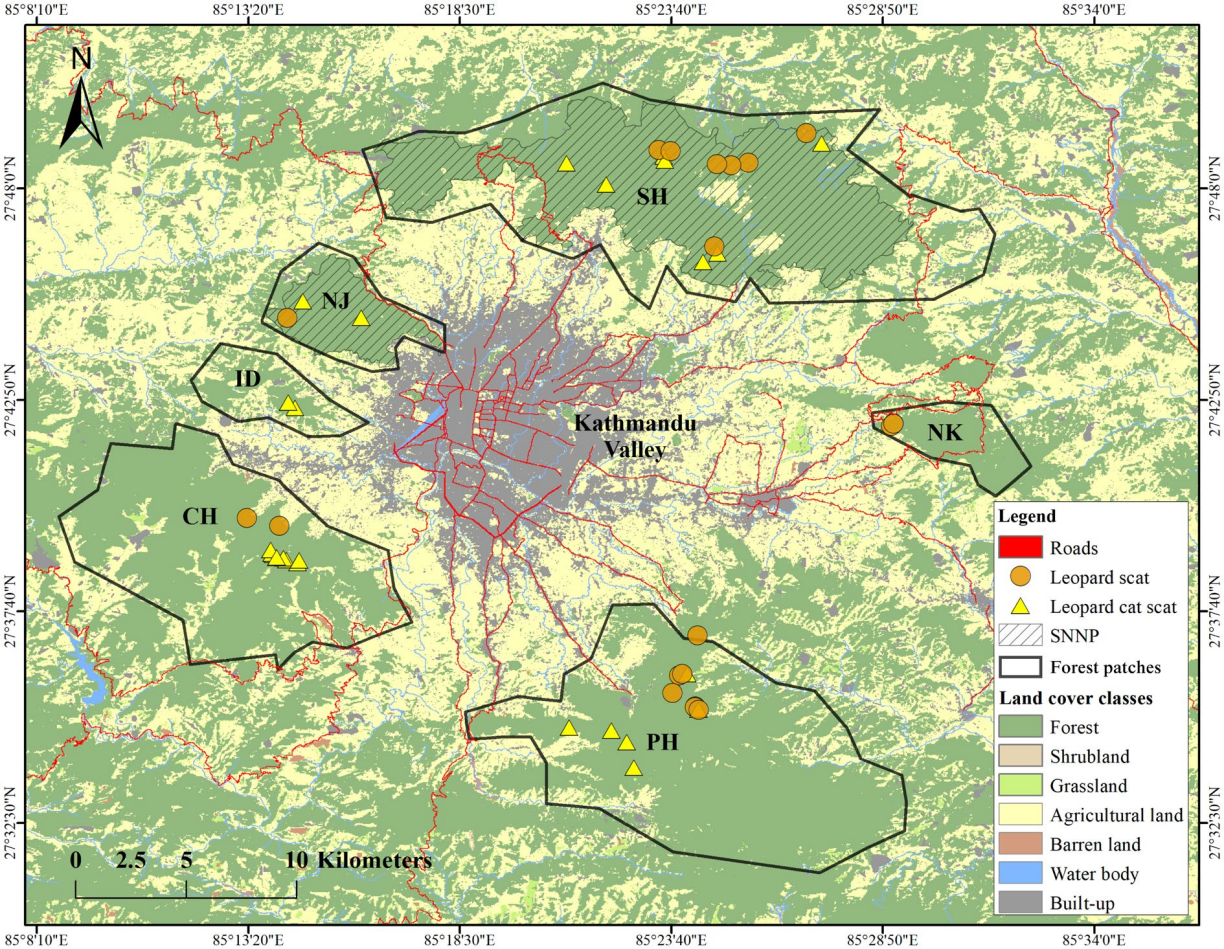
Spatial distribution of leopard and leopard cat fecal samples collected from the six noncontiguous forest patches (our study areas) surrounding the Kathmandu Valley, Nepal. Different land cover classes in the broader Kathmandu region are shown. The roads represent the major road network only, that is, primary and secondary roads (SNNP—Shivapuri‐Nagarjun National Park, SH—Shivapuri, NJ—Nagarjun, ID—Indradaha, CH—Chandragiri, PH—Phulchoki, and NK—Nagarkot).

We conducted the study in forested areas around the hills or mountains (1300–2700 m above sea level) of the Kathmandu Valley (approx. 800 km^2^, 27.5500° N to 27.8500° N and 85.1700° E to 85.5300° E, Figure 1), which lie in the mid‐hill topographic region of Nepal. It is a part of the Mahabharata range that parallels south of the Himalayan range. The valley bifurcates to form a bowl‐shaped basin and is the main historical financial hub of Nepal, housing the metropolitan areas of Kathmandu, Bhaktapur, and Lalitpur districts. It is one of the fastest‐urbanizing regions in South Asia (Ishtiaque, Shrestha, and Chhetri 2017). The mountain forests surrounding the valley include a national park and numerous community forests. Community forestry in Nepal began in the late 1970s to involve local people in forest management, aiming to improve livelihoods through the sustainable use of forest resources (Kanel 2004). We conducted scat surveys in six major forest patches (Shivapuri‐SH, Nagarjun‐NJ, Nagarkot‐NK, Phulchoki‐PH, Chandragiri‐CH, and Indradaha‐ID; Figure 1) for 1–2 weeks at each site during October 2018 to June 2019. All study sites except SH and NJ are managed under the community forestry system of Nepal. SH and NJ are two non‐adjoining sectors of the Shivapuri Nagarjun National Park (SNNP), which is the only national park lying completely within the mid‐hills topographic region of the country. Deciduous vegetation of subtropical and temperate climates dominates the natural habitat in the hill forests. The valley base stands at an elevation of approximately 1300 m above sea level (asl) and the peaks reach approximately 2800 m above sea level. The mean annual rainfall in the valley is about 1505 mm, with a prolonged monsoon season from June to September, and average annual temperatures fluctuate between 15°C and 29°C.

The Kathmandu Valley is one of Nepal's most populated urban centers with over 2 million people, housing approximately 10% of the country's population, even though it accounts for less than 1% of Nepal's total area (CBS 2012). Since 1989, urban areas have expanded by 412% due to the large‐scale rural‐to‐urban migration of people into the Valley (Ishtiaque, Shrestha, and Chhetri 2017; Rimal et al. 2017). Beyond the city fringes lie the cultivated lands, gradually transforming into peri‐urban settlements that have encroached into wildlife habitats and are now spreading close to the remaining forests of the surrounding hills (Haack and Rafter 2006; Ishtiaque, Shrestha, and Chhetri 2017). The expanding nature of human‐influenced disturbance encroaching into natural habitats has been one of the primary causes of rising human–wildlife conflict in this region and beyond (Acharya et al. 2017).

### 2.2 Field Collection and Predator Species Identification of Carnivore Scats

Most of our field sampling involved opportunistic searches for wild carnivore scats along ridgelines, hill slopes, ravines, and gullies across the mountain forests. We navigated a total of 243 km of transects through hiking trails, game trails, fire lines, and off‐road tracks. Each scat sample was collected in two tubes (2 mL and 50 mL). We swabbed the surface of the scats with sterile cotton swab sticks and stored them in 2 mL tubes containing DET buffer. Additionally, we collected a subsample (~2 g) in 50 mL tubes with DET buffer. The DET buffer was adapted from Wultsch et al. (2015) for fecal DNA preservation of felids. For scat age classification, we followed the method of Jackson and Hunter (1996), sampling only recent to fresh carnivore scats. Each sample tube was labeled with a unique identifier, and additional metadata (e.g., GPS coordinates, scat diameter, scat age) was recorded for each scat sample. In the field, the samples were stored at ambient temperature before being transported to the laboratory, where they were kept in a −20°C freezer.

We processed all fecal samples collected in 2 mL tubes for predator species identification. We turned around the swab, centrifuged at 5000 RPM for 1 min, followed by the elution of 600 μL supernatant in a clean collection tube, before continuing to the DNA extraction procedure using the QIAamp Stool Mini Kit (Qiagen, Germany) following the manufacturer's protocol. We included negative extraction controls during each extraction procedure.

To identify leopard samples, we developed a species‐specific PCR assay with primers designed to specifically target a ~200 bp region of the mitochondrial cytochrome *b* gene in leopards (Table S1). For primer design, reference sequences from leopards, other felids, and carnivores were retrieved from NCBI GenBank to identify regions uniquely conserved in leopard sequences. The primers were tested on samples alongside a leopard‐positive control, and leopard identification was confirmed by visualizing PCR bands through gel electrophoresis. To ensure the accuracy of the newly developed leopard‐specific PCR assay, a subset of leopard‐positive samples was further validated through Sanger sequencing.

For nonleopard fecal samples, we performed species identification using DNA barcoding by amplifying and sequencing a ~412 bp barcoding region of the cytochrome *b* gene using the universal primers mcb398‐f and mcb869‐r (Verma and Singh 2003). The resulting sequences were compared against the NCBI GenBank database using BLAST, applying a 97% nucleotide identity threshold to confirm species identity.

### 2.3 Prey Profiling Using DNA Metabarcoding

#### 2.3.1 Library Preparation and Next‐Generation Sequencing

We extracted whole DNA for all confirmed leopard and leopard cat scats from their replica 50 mL tube for vertebrate prey profiling. We vortexed the tube, followed by centrifuging at 5000 RPM for 1 min, and finally eluting 600 μL of supernatant in a clean Eppendorf tube. We then performed DNA extraction using the QIAamp Stool Mini Kit (Qiagen, Germany) following the manufacturer's protocol.

Next, we followed a two‐step PCR protocol described by Miya et al. (2015). In the first PCR, the vertebrate prey DNA contained in predator scats is amplified, whereas in the second PCR, unique oligo‐indices (barcodes) for each individual scat sample are attached to the amplified products of the first PCR, in order to allow for multiplexing the library. We amplified a short barcoding region (~100 bp) of the *12S rRNA* gene of vertebrate mtDNA using the universal vertebrate primers 12SV5F and 12SV5R (Riaz et al. 2011). Both primers were tagged with the Illumina overhang sequences to allow subsequent annealing of index primers incorporated with unique barcoding indices and Illumina sequencing flowcell adapters. We also included a blocking oligonucleotide that reduces amplification of host predator (leopard) DNA (Shehzad et al. 2015; see Table S1). This assay profiles all vertebrate prey DNA contained in predator scat.

For the first PCR, we used modified 12SV5 primers that had random hexamers and overhang oligonucleotide sequences in the 5′ region. The hexamers were included to enhance cluster separation during initial base call calibrations on the sequencer. We performed first‐round PCR in a total reaction volume of 25 μL containing 12.5 μL 2x KAPA HiFi HotStart ReadyMix (Kapa Biosystems, USA), 0.25 μL of each modified 12S gene‐specific primer (12SV5F/12SV5R), 2 μL template DNA, 6.25 μL blocking primer, and 3.75 μL H_2_O. We then performed initial denaturation of the PCR mastermix at 98°C for 3 min, followed by 35 cycles of 94°C for 30 s, 63.5°C for 30 s, 72°C for 30 s, and 72°C for 5 min.

For the second PCR, we indexed the first round PCR products of each sample using unique Illumina indices from Nextera XT Index Kit V2 (Illumina, USA) to allow multiplexing of the library during sequencing. The indexing primer contained complementary oligonucleotides that enhanced annealing to the overhang sequence in the first round PCR product, along with unique indices and Illumina sequencing adapters. We performed the second PCR in a total reaction volume of 50 μL containing 25 μL 2x KAPA HiFi HotStart Ready Mix, 5 μL of forward and reverse Nextera XT Index primers, 5 μL template (first PCR product), and 10 μL H_2_O. We performed initial denaturation of the PCR mastermix at 95°C for 3 min, followed by 8 cycles of 95°C for 30 s, 55°C for 30 s, 72°C for 30 s, and 72°C for 5 min.

We cleaned the final amplicons using AMPure XP magnetic beads. We quantified libraries with the Qubit dsDNA HS assay kit (Thermo Fisher Scientific, USA), followed by normalization (4 nM) for loading in the sequencer. We performed paired‐end (2× 150 bp) sequencing (loading concentration 10 pM) on an Illumina MiSeq instrument using a 300‐cycle MiSeq Reagent Kit v2 (Illumina Inc., USA) that generated demultiplexed fastq sequence reads of each sample by MiSeq Reporter v2.6.4 application.

#### 2.3.2 Sequence Data Processing and Taxonomic Identification

The quality of the raw DNA sequence reads (fastq files) from Illumina MiSeq (Illumina Inc., USA) was assessed using FastQC v0.11.9 (Andrews et al. 2010). Subsequently, Trimmomatic v0.39 (Bolger, Lohse, and Usadel 2014) was employed to remove adapter sequences, followed by trimming reads shorter than 95 bp and those with low quality (*Q*‐score < 20). Using the metabarcoding pipeline QIIME2 v2021.4 (Bolyen et al. 2019), paired‐end reads were further refined to eliminate various sequencing artifacts and correct potential PCR and sequencing errors based on error models. This denoising step, performed using the DADA2 plugin, involves removing sequencing errors and low‐quality bases, merging paired‐end reads, and identifying and eliminating chimeric sequences. This process generates clean, unique sequences known as amplicon sequence variants (ASVs).

Only ASVs belonging to the target taxa (i.e., vertebrate) were used for further analysis. Nontarget ASVs were filtered out using BLAST similarity searches against the Vertebrate 12S reference database, compiled and curated from NCBI GenBank. The remaining target ASVs were classified to their taxonomy levels using a consensus BLAST algorithm within QIIME2's feature‐classifier function. The consensus taxonomy was assigned from among the maximum 50 hits returned by BLAST, with a minimum of 51% consensus required for classification. BLAST analysis parameters included a query coverage of 85% and a nucleotide percentage identity of 97%. The ASVs were clustered into operational taxonomic units (OTUs), which provide a closer relation to the species concept and are more robust than the traditional approach of clustering OTUs before taxonomic assignment.

Based on the consensus taxonomy classification, the OTUs were assigned to various taxonomic levels, including species, genus, family, and so on. The OTU table was further filtered using the minimum read threshold derived from the negative control sample included in the sequencing run. We calculated relative abundance for all samples based on taxa and created a presence/absence table using the criterion that the prey would be assigned as present if the relative abundance was greater than 1%, thus further filtering out rare sequencing artifacts. Additionally, OTUs classified only up to the family level and those that remained unassigned were excluded from downstream analysis to improve interpretability.

#### 2.3.3 Statistical Analyses of Dietary Data

We also reported the percent occurrence (PO) for each prey item to determine the abundance of each item in the diet of the leopard and leopard cat. We defined it as *PO = s*/*N*, where *s* is the number of occurrences of specific prey items and *N* is the total occurrences of all prey items in all scat samples of the leopard and leopard cats (Khatoon et al. 2019). PO reveals how important a prey item is in the diet, but it often overestimates the importance of small prey because it measures the frequency with which prey appears in a predator's diet but does not account for the biomass contribution (Ackerman, Lindzey, and Henker 1984). Thus, we also calculated the percentage of scats containing each prey species, that is, frequency of occurrence (FO) as an additional metric.

We calculated the dietary niche breadth of the two carnivores using the standardized Levins index. It is measured as *Bsta =* (1/*∑pi*2)−1/(*n*‐1), where *pi* is the relative frequency of prey items consumed by predator *p* and *n* is the total number of prey items ingested (Levins 1968; Marshall and Elliott 1997; Briers‐Louw and Leslie 2020; Palei et al. 2022). Values closer to zero indicate specialist predators, and those closer to one indicate generalist species.

We then conducted principal component analysis (PCA) in R v3.6.2 (R Core Team 2019) implemented in R Studio v1.2 (RStudioTeam 2019) using the presence/absence data of the prey items in the carnivore scats and grouped them into various taxa, mostly based on the order and class of the prey items. PCA is an eigenvector‐based multivariate analysis that creates a simplified summary of existing patterns in the data set. Linear combinations of the original variables are produced, thus reducing the dimensionality of the original multivariate data and retaining only the most important information (Abdi and Williams 2010). We conducted multiple linear regression with the first principal component scores as the response variable and forest patch and predator species as two explanatory variables. Using the same response and explanatory variables from the gplots package v3.0.1.1 (Warnes et al. 2019) in R, we used the plotCI function to visualize the mean reliance of the two carnivore species on domestic prey items in the region by plotting a 95% confidence interval plot. We defined reliance on domestic prey items based on how commonly domestic prey items occurred in leopard and leopard cat scats.

### 2.4 Geospatial Analyses of Carnivore Feeding Habits

#### 2.4.1 Land Cover Mapping

We represented the land cover classes in the study area at a spatial resolution of 30 m using the Transverse Mercator projection in ArcGIS v10.7.1 (ESRI; Redlands, CA, USA). The most recent mapping of land cover in Nepal was conducted by Uddin et al. (2015) for the International Centre for Integrated Mountain Development (ICIMOD). In order to update the map, we added an additional built‐up area as mapped by the Global Urban Footprint project (Esch et al. 2017) and as mapped on OpenStreetMap. We define ‘built‐up’ area as any space where human development has occurred, including the urban and rural fabric, except for agricultural areas. Similarly, we merged the OpenStreetMap waterways raster with the ICIMOD 2010 water layer. We then overlaid the above updated built‐up and water layers with the ICIMOD 2010 land cover data set. This revised land cover data set gave us a more up–to‐date presentation of the land cover classes in the region.

#### 2.4.2 Assessing Habitat Disturbance

To quantify the extent of habitat disturbance around the carnivore scat collection points, we estimated the proportions of the major land cover classes, namely forest, agricultural (farmlands mostly containing food crops like grains, potato, and mustard), and built‐up area within five buffer radii (1000/1500/2000/3500/4500 m). We chose these buffer radii based on the average home range sizes of leopards and leopard cats in human‐dominated landscapes across Asia (Miller 2011; Mohamed et al. 2013; Odden et al. 2014). Furthermore, to closely examine the habitats that the two carnivores occupy, we calculated the distance of each scat from the nearest forest edge, that is, the boundary between the forest and any other land‐use type.

We used FRAGSTATS v4.2 (McGarigal and Ene 2015) to determine the extent of forest fragmentation in the study region. Class‐level indices in FRAGSTATS can quantify the extent of habitat fragmentation for each of the land cover classes, especially the forest class (McGarigal and Marks 1995). We chose six class metrics (Table S2) based on previous studies that similarly assessed landscape fragmentation (Southworth, Munroe, and Nagendra 2004; Miyamoto and Sano 2008; Midha and Mathur 2010). We adopted the eight‐neighborhood cell criterion for defining the land cover class patches and fixed an edge depth of 100 m to assess the edge density and mean core area, based on previous research (Laurance 2000; Broadbent et al. 2008; Midha and Mathur 2010).

We saved the major land cover raster layers as GeoTIFF files and used them as input for the fragmentation analysis. The fragmentation metrics were computed at three scales—the entire study area, the six major forest patches, and 1000 m around the scat collection locations. We chose 1000 m as the neighborhood as it showed the most statistically significant difference from the other buffer radii (independent two‐sample test, *p* value = < 0.05).

Next, we used the values obtained for each class‐level metric run for the corresponding forest patches to determine the degree of habitat fragmentation in the forest patches located 1000 m around the scat collection locations. We ranked the values of the six metrics and calculated a median from the six metric ranks for each forest patch. We classified the lowest scoring medians as areas with low fragmentation, middle values as moderately fragmented, and highest scoring medians as highly fragmented forests.

To assess the effect of habitat disturbance in the study area on the diets of the two carnivores, we conducted multiple linear regression. We used (1) the proportion of forests 1000 m around the scats, (2) the distance of scats from the nearest forest edge, and (3) the degree of forest fragmentation measured 1000 m around the scats as the explanatory variables and the first principal component scores from the PCA as the response variable.

We also analyzed the proportion of forest, agricultural, and built‐up areas located 1000 m around the carnivore scats containing cattle ( *Bos taurus* ) and those without cattle. The purpose of this analysis was to understand the land cover type prevalent in the areas where the scats were found. We chose to focus the analysis on cattle since it was the most abundant domestic prey species in leopards' diets.

#### 2.4.3 Potential Human‐Leopard Conflict Hotspots

The concept of Wildland–Urban Interface identifies heterogeneous spaces that are often more susceptible to incidences of wildfires, habitat fragmentation, biodiversity decline (Radeloff et al. 2005; Massada et al. 2009; Biasi et al. 2015) and in this case, human‐carnivore conflict. We therefore used Forest–Built‐up and Forest–Agricultural interfaces to represent areas that are likely to be more susceptible to human–leopard conflict.

We used ArcGIS v10.7.1 (ESRI; Redlands, CA, USA) to create binary layers of the forest, agriculture, and built‐up classes. In the Focal Statistics tool, we individually selected the three binary layers and adopted a circular neighborhood of 150 m, based on the average distance of leopard activity away from forests (Dahat 2013; Kulkarni 2016; Viollaz 2016; Reporter 2019; Joseph 2020). In Raster Calculator, we used the multiplication operator that multiplies the values of raster layers on a cell‐by‐cell basis to multiply the forest and agricultural binary layers and to multiply the forest and built‐up binary layers. We thus generated the Forest–Agricultural Interface and Forest–Built‐up Interface. We then produced the Forest–Agricultural–Built‐up interface by multiplying (i.e., intersecting) the Forest–Agricultural and Forest–Built‐up interfaces.

## 3 Results

### 3.1 Species Identification

We collected 57 scat samples across the hills of the Kathmandu Valley, of which 20 (35%) samples were from SNNP (SH, *n* = 14; NJ, *n* = 6), whereas the remaining 37 (65%) were from ID (*n* = 2), CH (*n* = 14), PH (*n* = 18), and NK (*n* = 3) forests. Among these, we could not identify two samples due to PCR failure (these were degraded samples collected during the monsoon period). Out of the remaining 55 samples, we identified 25 (45.5%) as leopards. Leopard scats had an average of 3.3 cm (range 2.8–4 cm) diameter, in addition to key felid scat features like segmented texture and a tapered end at one side. Among the remaining samples that were not leopards, we identified 26 (47.3%) as leopard cats. Scats of leopard cats had an average diameter of 1.7 cm (range 1–2.5 cm). Last, the remainders (7.2%) were identified as yellow‐throated marten ( *Martes flavigula* , *n* = 2) and large Indian civet ( *Viverra zibetha* , *n* = 2).

Among the 25 scats of leopard, we identified 10 from PH, seven from SH, four from CH, three from NK, and one from NJ. We also detected leopard scats in ID, but we did not collect them as they were very old (more than a month) and dried out. Similarly, among the 26 scats of leopard cats, we identified nine from CH, seven from SH, six from PH, and two each from ID and NJ. The two scats of yellow‐throated marten and large Indian civet were collected from PH and NJ, respectively.

### 3.2 Diet Profiles of Leopards and Leopard Cats

Out of 25 leopard scats, five did not pass the quality control steps during library preparation and were rejected from further processing. The remaining 46 scat samples (leopard, *n* = 20 and leopard cat, *n* = 26) generated a total of 4,678,852 reads (mean per sample: 101,714; range: 18,511–207,627 reads) using a DNA metabarcoding approach.

From the 20 leopard scat samples processed for diet analysis, we identified 13 vertebrate prey taxa and a total of 37 prey items (Table 1). Based on the percent occurrence of each food item per total food items, we found that the leopard diet was mostly dominated by ungulates (PO = 59.5%) which mainly consisted of cattle (PO = 24.3%) and barking deer (*Muntiacus vaginalis*, PO = 16.2%). Birds, mainly represented by domestic fowl or chicken, also constituted a substantial portion (PO = 18.9%) of the leopard diet. This was followed by rodents (PO = 17%), which consisted mainly of mice (*Mus* spp) and porcupines (*Hystrix* spp.). Occurring in 16 of 20 scats (80% of scats), domestic prey comprised nearly half (51.4%) of the leopard diet, whereas wild animals occurred in 12 of 20 scats (49%).

**TABLE 1:** Diet composition of leopards in the forests around the Kathmandu Valley. Here, %PO is the percent occurrence of the prey in the scats and %FO is the percentage of scats containing each prey species.

Prey type/order/species	%PO *n* = 20	%FO *n* = 20	Approximate prey weight (kg)
Domestic			
Ungulates			
Cattle ( *Bos taurus* )	24.32	45	167
Goat ( *Capra hircus* )	8.11	15	26
Sheep ( *Ovis aries* )	2.70	5	27
Carnivores			
Dog ( *Canis lupus familiaris* )	2.70	5	12
Birds			
Domestic fowl ( *Gallus gallus* )	13.51	25	0.78
Wild			
Ungulates			
Barking deer (*Muntiacus vaginalis*)	16.22	30	18
Sambar deer ( *Rusa unicolor* )	5.41	10	212
Wild boar ( *Sus scrofa* )	2.70	5	38
Primates			
Rhesus macaque ( *Macaca mulatta* )	2.70	5	6
Birds			
Eagle (*Aquila* spp)	2.70	5	3
Kalij pheasant ( *Lophura leucomelanos* )	2.70	5	0.89
Rodents			
Mouse (*Mus* spp.)	13.51	25	0.5
Porcupine (*Hystrix* spp.)	2.70	5	1.5

Of the 26 scat samples of leopard cats processed for diet analysis, we identified 15 prey taxa among 76 total prey items (Table 2). We found that the leopard cat diet was primarily dominated by rodents (PO = 76.3%) in terms of percent occurrence, with at least six genera identified. *Niviventer* spp. (PO = 25%) followed by *Mus* spp. and *Rattus* spp., both PO = 21.1%, were the most important prey in their diet. Other prey, such as ungulates (PO = 10.5%), birds (PO = 5.3%), shrews, and fishes (both PO = 3.9%), also occurred in their diet. Domestic prey only occurred in 1 of 26 (1.3%) leopard cat scats, whereas wild animals occurred in 25 of 26 (98.6%) scats, and therefore, wild prey dominated the leopard cat diet.

**TABLE 2:** Diet composition of leopard cats in the forests around the Kathmandu Valley. Here, %PO is the percent occurrence of the prey in the scats, and %FO is the percentage of scats containing each prey species.

Prey type/order/species	%PO *n* = 26	%FO *n* = 26	Approximate prey weight (kg)
Domestic			
Ungulates			
Cattle ( *Bos taurus* )	1.32	3.85	167
Wild			
Rodents			
Bandicoot rat (*Bandicota* spp.)	2.63	7.69	0.58
Giant pouched rat (*Cricetomys* spp.)	3.95	11.54	1.25
Mouse (*Mus* spp.)	21.05	61.54	0.5
Niviventer rat (*Niviventer* spp.)	25.00	73.08	0.08
Rattus rat (*Rattus* spp.)	21.05	61.54	0.15
Giant flying squirrel (*Petaurista* spp.)	2.63	7.69	1.65
Shrew			
Brown‐toothed shrew (*Episoriculus* spp.)	3.95	11.54	0.01
Birds			
Hill partridge ( *Arborophila torqueola* )	1.32	3.85	0.31
Wheatear (*Oenanthe* spp.)	1.32	3.85	0.02
Puff‐throated babbler ( *Pellorneum ruficeps* )	1.32	3.85	0.03
Kalij pheasant ( *Lophura leucomelanos* )	1.32	3.85	0.89
Fish			
Carp fish (*Ptychidio* spp.)	1.32	3.85	2.5
Snowtrout (*Schizothorax* spp.)	2.63	7.69	2.5
Ungulates			
Barking deer (*Muntiacus vaginalis*)	9.21	26.92	18

**FIGURE 2:**
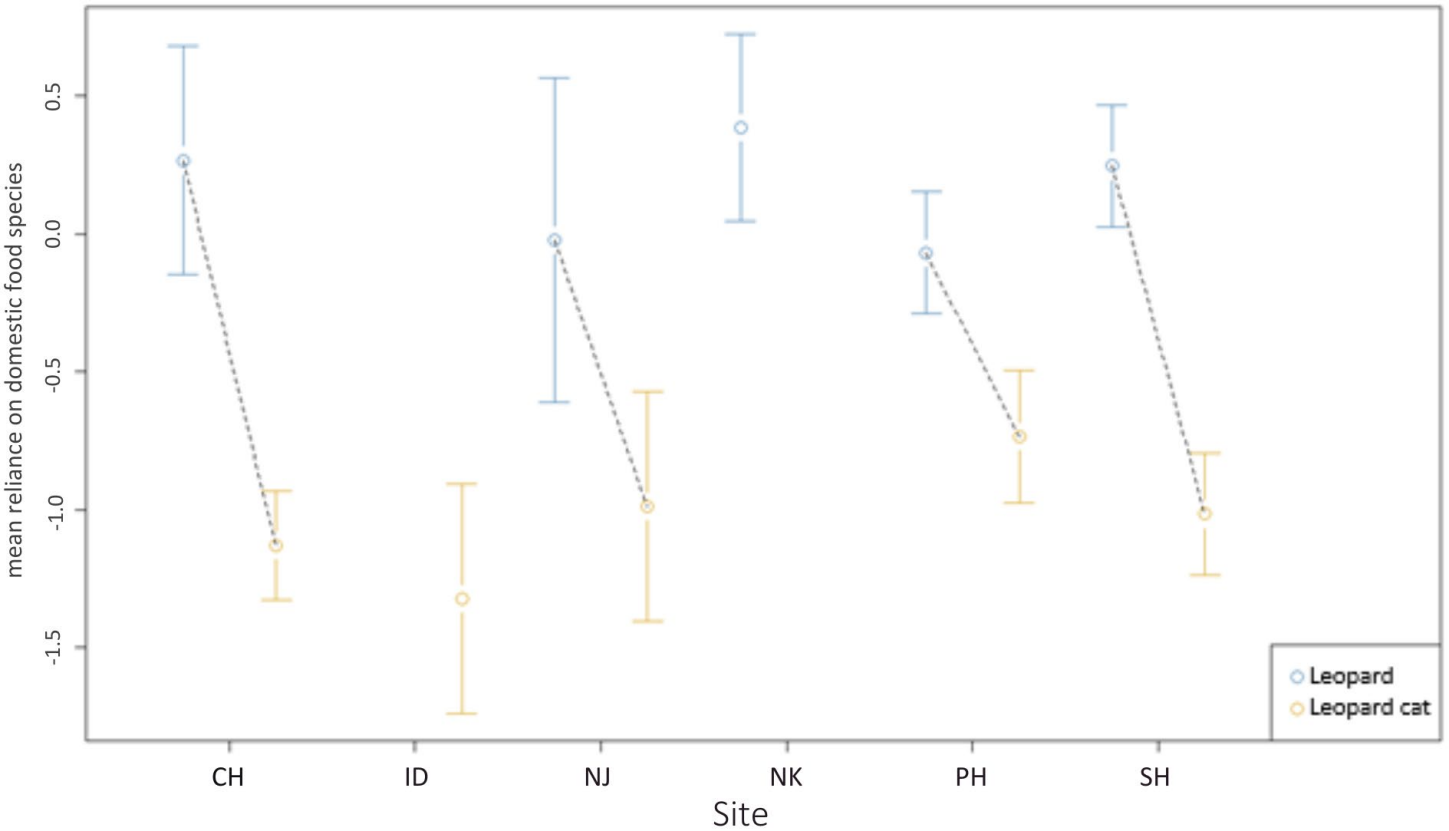
Reliance of leopards and leopard cats on domestic prey across the surveyed forests of the Kathmandu Valley, Nepal. The positive and negative values on the y‐axis signify high and low reliance of the carnivores on domestic prey species, respectively. Scats of leopards and leopard cats were not collected from ID and NK, respectively. (CH—Chandragiri, ID—Indradaha, NJ—Nagarjun, NK—Nagarkot, PH—Phulchoki, and SH—Shivapuri).

Levin's standardized dietary niche breadth of leopards was relatively wider (Standardized Levins Index = 0.18, *n* = 20) than that of leopard cats (Standardized Levins Index = 0.07, *n* = 26). We found leopards to be more reliant on domestic prey compared with leopard cats across all the surveyed forests (Figure 2). The multiple regression analysis revealed that the two predictor variables (specific forests and predator species) explained 80% of the variance for the response variable (the first principal component scores) (*R* ^2^ = 0.80, *F* (6.39) = 26.47 *p* = 2.579e‐12). The degree of reliance on domestic prey was different both between predators (two‐way ANOVA, *F* = 118.877, *N* = 46, df = 1, *p* = 5.868e‐13) and between forest patches (two‐way ANOVA, *F* = 13.588, *N* = 46, df = 5, *p* = 1.800e‐07). We also found the interaction term between forest patches and predators to be statistically significant (two‐way ANOVA, *F* = 3.292, df = 3, *p* = 0.03143), which further explains that leopards relied more on domestic prey across all forests.

### 3.3 Extent of Habitat Fragmentation

**FIGURE 3:**
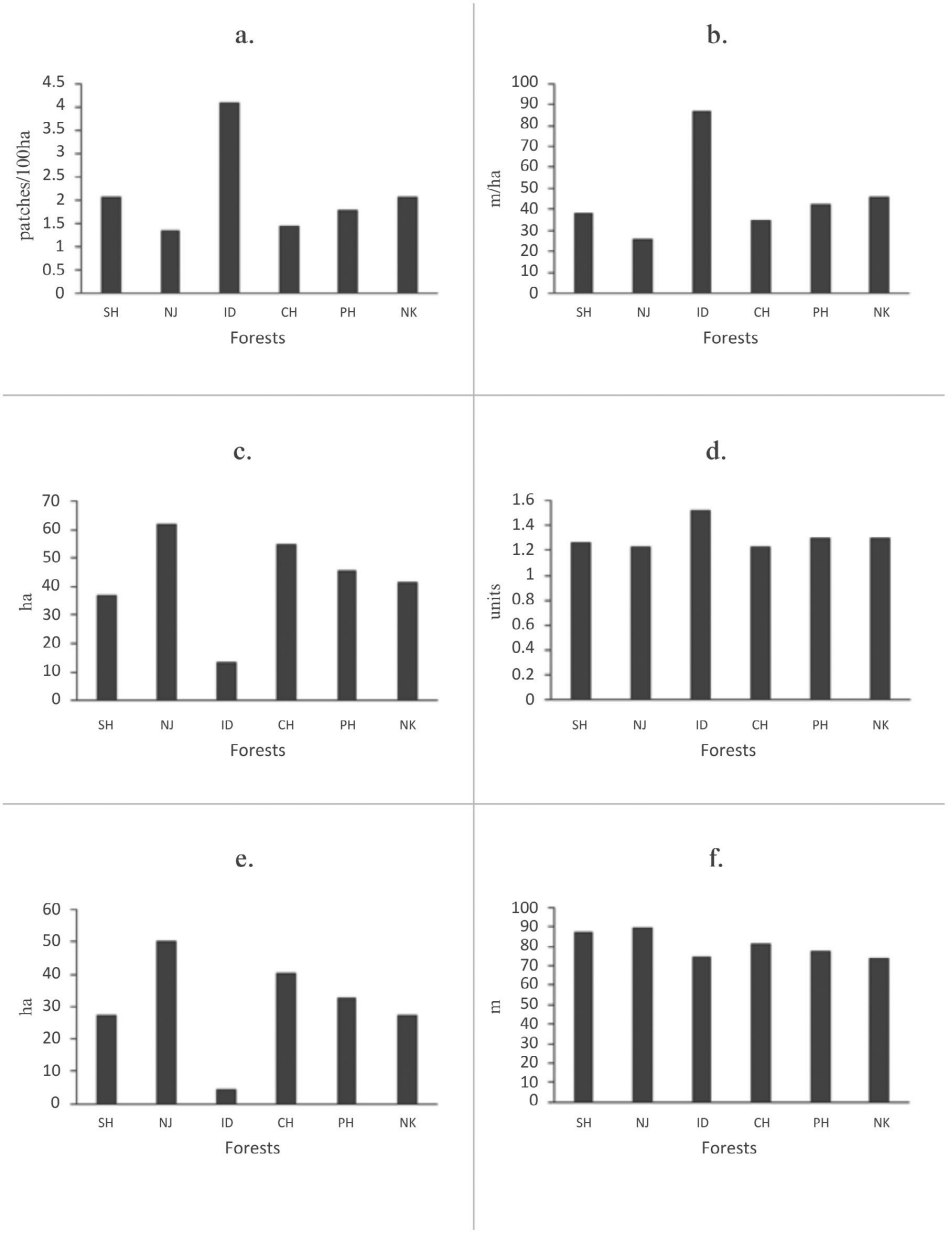
Fragmentation class metric values for the six forests. (a) Patch density, (b) edge density, (c) mean patch area, (d) mean shape index, (e) mean core area, (f) mean Euclidean nearest neighbor distance (SH—Shivapuri, NJ—Nagarjun, ID—Indradaha, CH—Chandragiri, PH—Phulchoki, and NK—Nagarkot).

Of the total study area, 51% consisted of human‐used land, that is, 30% agricultural and 20.8% built‐up (Figure 1). However, only 46% of the land was covered by forests, most of which were fragmented. Shrubland, grassland, barren land, and water bodies comprised the remaining 2.8% of the land cover. Given below are the results of the forest fragmentation analysis (Table 3 and Figure 3). Further details of the metrics are also provided in Tables S2 and S3.

**TABLE 3:** Level of forest fragmentation based on 1000 m neighborhood around carnivore scat collection points (*n* = 46) and the quantities of domestic and wild prey eaten by the predators. The median rank ranges (1–46; lowest to highest fragmentation) were derived by ranking the fragmentation metric values of each surveyed forest followed by calculating the median from the ranks obtained. The median rank for each forest area is the median calculated from the median rank range for the corresponding forest.

Forest	Fragmentation level	Median rank range	Median rank	Leopard diet (*n* = 20)			Leopard cat diet (*n* = 26)		
				No. of scat	No. of domestic prey	No. of wild prey	No. of scat	No. of domestic prey	No. of wild prey
Shivapuri	Low–moderate	1 to 39	8.5	7	8	6	7	0	18
Nagarjun	Moderate	11 to 39	11	1	0	1	2	0	5
Chandragiri	Moderate	11 to 39	22	2	3	1	9	1	33
Phulchoki	Moderate	11 to 39	28.5	7	5	9	6	0	13
Indradaha	High	40 to 46	41.5	NA	NA	NA	2	0	6
Nagarkot	High	40 to 46	45	3	3	1	NA	NA	NA

*Note:* NA—no leopard and leopard cat scats were collected from Indradaha and Nagarkot, respectively.

#### 3.3.1 Patch Density and Mean Patch Area

Our findings of the patch density of the entire study area and of the six forests suggest that forest land had been subdivided into many patches. We found that ID had the lowest mean patch area (13.52 ha), whereas NJ and CH had the highest mean patch areas with corresponding low patch densities; this implies that ID was most fragmented.

#### 3.3.2 Edge Density, Mean Shape Index, and Mean Core Area

ID obtained the highest edge density, whereas the lowest value was obtained by NJ. All the surveyed forests had irregular shapes, and their mean shape index values were greater than one which implies fragmentation. Except for ID, we found that all the other surveyed forests recorded a mean core area > 50% of the corresponding mean patch area. As the amount of core area represented is affected by the shape, our findings suggested that the edge effect was relatively low in the five forests surveyed, with ID as an exception.

#### 3.3.3 Mean Euclidean Nearest Neighbor Distance

NJ had the greatest distance, which indicates that its forest patches were located relatively farther away from each other in comparison to the other forests examined. The nearest neighbor distance of the forests in the entire study area was 99 m; this explains that the forest patches across the study area were not contiguous with one another.

The fragmentation metric values obtained for the forest class 1000 m around the 46 carnivore scat collection locations were consistent with the above findings (Table 3). Overall, all six major forest patches show signs of varying degrees of habitat fragmentation, especially ID, where the forest was most fragmented (Table 3).

### 3.4 Effect of Habitat Disturbance on the Carnivores' Feeding Ecology

Based on multiple linear regression, the three explanatory variables of habitat disturbance (proportion of forests 1000 m around the scats, distance of scats from the nearest forest edge, and level of forest fragmentation 1000 m around the scats) explained 32% of the variance for the response variable (the first principal component scores) (*R* ^2^ = 0.32, *F* (3,42) = 6.59, *p* = 0.0009). We found no statistically significant effect of habitat disturbance on the dietary habits of the two carnivore species (Table S4).

**FIGURE 4:**
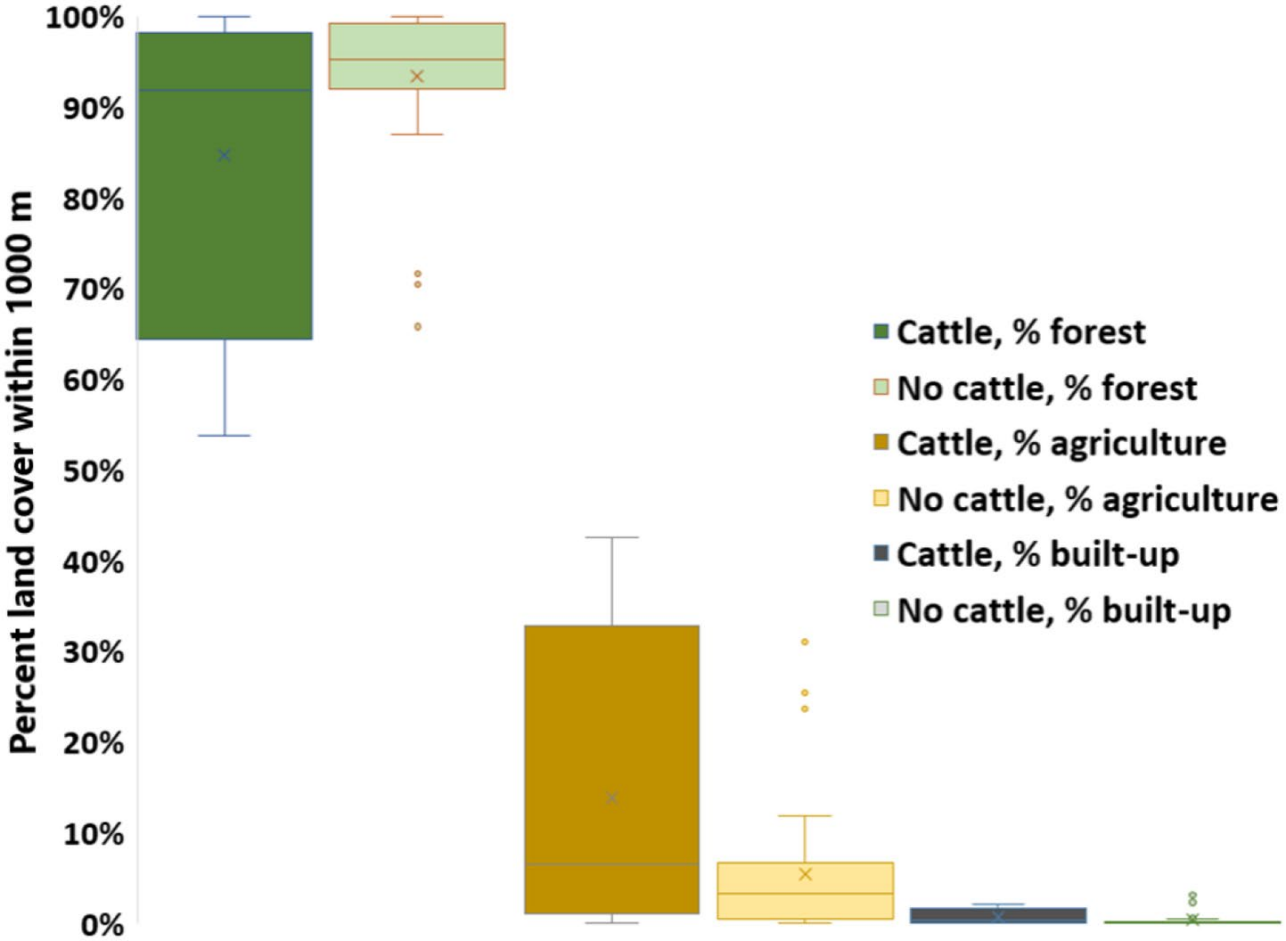
Percent land cover within 1000 m around carnivore scats containing cattle and those without cattle.

However, we found a statistically significant difference between scats that had cattle (*n* = 10) and those without cattle (*n* = 36) when the percent cover of the three major land cover classes within a radius of 1000 m around the scats was analyzed (Figure 4). Scats with cattle were located within areas with less forest cover (84.7% compared with scats without cattle 93.3%; *p* < 0.05), more agricultural area (13.8% compared with scats without cattle 5.4%; *p* < 0.05), and more built‐up area (0.7% compared with scats without cattle 0.3%, however, this was statistically insignificant).

### 3.5 Interfaces Predicting Potential Human–Leopard Conflict Hotspots

**FIGURE 5:**
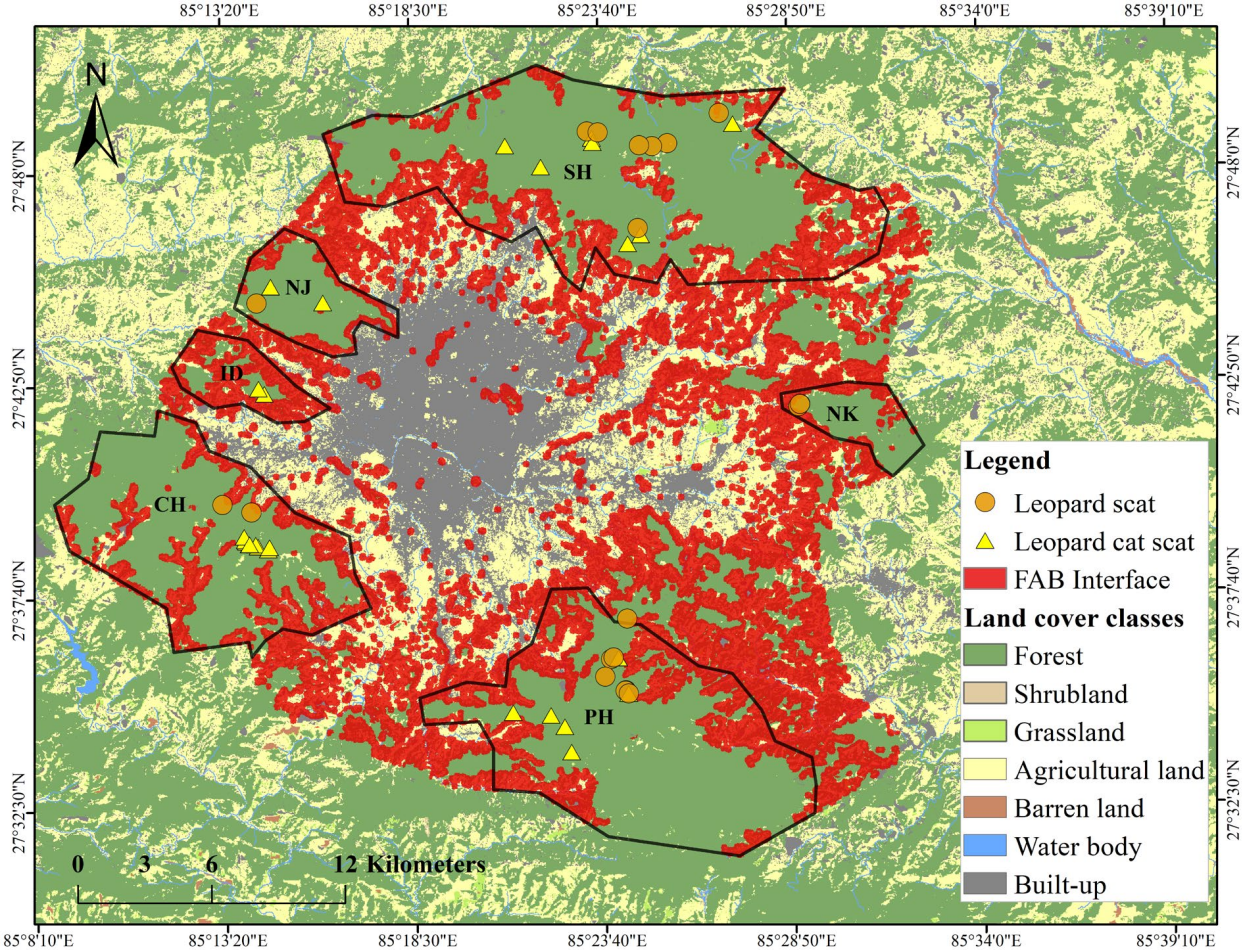
Forest–Agricultural–Built‐up (FAB) interface (i.e., areas predicted to have higher frequency of human–leopard encounters in the landscape) in the Kathmandu Valley, Nepal. Star‐marked scats symbolize carnivore scats with cattle (*n* = 10; leopard = 9, leopard cat = 1) (SH—Shivapuri, NJ—Nagarjun, ID—Indradaha, CH—Chandragiri, PH—Phulchoki, and NK—Nagarkot).

The Forest–Built‐up Interface made up 41% (39,397 ha) and the Forest‐Agricultural Interface comprised 48% (45,301 ha) of the total landscape. Based on the concept of Wildland‐Urban Interface, the Forest–Agricultural–Built‐up Interface representing the overall areas likely most susceptible to human–leopard conflict made up 39% (37,525 ha) of the total study area (Figure 5). These heterogeneous areas were identified along the outskirt or peri‐urban regions of the Kathmandu Valley and are the potential hotspot regions where encounters between humans and leopards are high.

## 4 Discussion

In this study, we employed DNA metabarcoding to gain insights into the feeding behaviors of two felid species inhabiting the forests surrounding an urbanized area. Our focus was primarily on the significance of domestic prey in the diet of leopards and leopard cats within a human‐dominated landscape, while also examining the relationship between the dietary habits of urban leopards, urbanization, habitat fragmentation, and conflicts in the Kathmandu Valley. To achieve this, we utilized a DNA‐based method to noninvasively identify predator species from scat samples. The metabarcoding approach enabled us to profile the vertebrate prey taxa more accurately compared to previous studies, which predominantly relied on traditional microhistological techniques (Achyut and Kreigenhofer 2009; Koirala et al. 2012; Kandel, Lamichhane, and Subedi 2020). In ecosystems where multiple carnivores cooccur and compete for similar resources, relying solely on traditional scat identification techniques may result in misidentification and misinterpretation of both predator and diet profiles. Our field‐collected scats were identified as belonging to leopards, leopard cats, martens, and civets using DNA‐based species identification. Habitat fragmentation resulting from rapid urbanization in Kathmandu (Ishtiaque, Shrestha, and Chhetri 2017; Rimal et al. 2017), which appears to be haphazard and unplanned, has impacted biodiversity, especially in the context of carnivore‐associated conflicts such as those observed with leopards in the area. Our study delved into this complex interplay by integrating carnivore diet data with geospatial analysis, aiming to elucidate the correlation between various geographic features and carnivore feeding ecology. This comprehensive approach allowed us to evaluate the profound effects of habitat fragmentation on the foraging behaviors of two carnivore species in the urban ecosystem of Kathmandu.

### 4.1 Urbanization Influences the Human–Leopard Conflicts in Kathmandu

Leopards are generalist predators, adept at subsisting on various prey in their habitat, allowing them to adapt to changing environments (Hayward et al. 2006). Our study showcases that despite the low Levin's dietary niche breadth i.e., far from 1, which indicates a high dietary specialisation, the leopards in the Kathmandu Valley have a wide variety of prey species, suggestive of the generalist feeding habits usually associated with leopards. Large felids like leopards are known to specialize in preying on medium‐ to large‐sized vertebrate species, often feeding for multiple days on a single prey animal to conserve energy expenditure during consecutive kills (Elbroch et al. 2017; Barry et al. 2019). Such might also be the case in the Kathmandu Valley, where a single dead, large‐sized animal like a cow can provide multiple, entire meals to leopards in the area. Despite the relative generalist feeding habits of leopards in our study area, we did observe that the majority of the leopard's prey consisted of medium‐ and large‐sized ungulates, including barking deer (wild prey) and cattle (domestic prey) (Table 1). While leopards are known to prey on livestock, it remains uncertain whether they actively hunt or scavenge stray cattle or domesticated cattle owned by humans.

The reliance on domestic prey was evident across all study sites, except for ID, from which we did not have any leopard samples (Figure 2). ID, characterized by high levels of fragmentation (Table 3 and Figure 3), posed challenges in finding fresh samples due to rapid land‐use changes, such as deforestation, land plotting, road cutting, and urban development activities that occurred during the study period. Despite these challenges, we discovered a few old scats in the ID forest, which may indicate that leopards were temporarily displaced due to the high level of disturbance. However, we did not collect these samples due to the expected DNA degradation in such old‐aged samples. Most of our study sites are managed under the community forest system, which is not federally protected, thereby allowing various levels of land‐use or developmental activities within these forest patches. Interestingly, in the SH sector of SNNP, our study revealed domestic prey reliance among leopards, which was similarly exhibited in CH, NJ, and NK (Figure 2). Leopards mainly depend on prey availability and mostly prefer medium‐sized ungulates (10–40 kg) where available, aligning with their generalist feeding habits (Hayward et al. 2006; Stein, Bourquin, and McNutt 2015). The evident reliance of leopards on domestic prey species in SH can be attributed to the presence of longstanding villages within SH, where livestock such as cattle, goats ( *Capra hircus* ), and chickens ( *Gallus gallus* ) are commonly raised and graze alongside wild prey species in SNNP. The adaptability of leopards to domestic prey, including cattle, goats, dogs, and poultry (chickens), in Kathmandu's evolving urban landscape likely contributes to increased encounters and conflicts with humans, which has been outlined clearly in a semi‐structured survey by Bista et al. (2021). This adaptability‐driven human–leopard interaction emphasizes the need for informed management strategies to address the conflicts effectively.

Building upon prior observations that briefly touch on the rise of human–leopard conflict incidents may be due to habitat fragmentation in Kathmandu (Pokharel 2015), our findings shed light on how this fragmentation over recent decades due to urbanization drives changes in carnivore diets. Specifically, we observed a trend where leopards increasingly preyed on cattle, particularly in areas characterized by high levels of heterogeneous landscape features, that is, a mix of forest, agricultural, and built‐up land (Figure 4). This shift toward domestic prey aligns with the general pattern seen in human‐dominated regions (Kshettry, Vaidyanathan, and Athreya 2018; Kumbhojkar et al. 2021), where the abundance of domestic animals often surpasses that of wild prey (Schaller 1983). A study even found only domestic prey in the leopard diet, where there was probably no wild prey present in the forests (Shehzad et al. 2015). Kathmandu also faces a unique problem with its stray cattle population, mainly due to domestic cows being protected under federal law against slaughter for meat. This protection leads to people abandoning old‐aged cows and male calves (unfit for dairy purposes) in the outskirts of the valley. Much of the stray cattle may have become the staple diet for leopards, as evidenced by our study samples (Table 1).

The ongoing transformation of land use in Kathmandu Valley, coupled with such anthropogenic factors allowing ample availability of domestic prey such as stray cattle, likely contributes to the decline in wild prey species like barking deer. This decline is due to the degradation of forest areas and resource competition with domestic animals. As a result, resilient predators like leopards are forced to rely more on domestic prey, which are abundant and lack the anti‐predator adaptations of their wild counterparts (Geffroy et al. 2020), making them easier targets for predation. Although the specific population and density of wild prey in Kathmandu's forests are unknown, the prevalence of cattle in the leopard's diet provides valuable insights into these ecological dynamics. Overall, our study underscores the intricate relationship between habitat fragmentation, driven by urbanization, and its cascading effects on carnivore feeding behaviors and biodiversity dynamics in urban ecosystems like Kathmandu. In addition to stray cattle, Kathmandu also harbors an abundant population of stray dogs mainly along the dense city areas (Kakati 2012), which constitute an important prey source for urban leopards elsewhere (Surve et al. 2015). However, our analysis detected dog remains in only one leopard scat sample among 20 analyzed. This limited detection could be attributed to our sample size and the fact that our sampling sites were primarily located near the edges of forest areas along peri‐urban regions on the outskirts of the cities, dissociated from densely populated human settlement regions where urban leopards are known to frequently hunt and prey upon dogs. Future research endeavors in Kathmandu should consider expanding sampling efforts to encompass the forested regions, peri‐urban, and urban areas. Such an approach would provide a more comprehensive understanding of the dietary preferences and profiles of urban leopards, particularly in relation to their interactions with domestic animals like stray dogs and cattle. This expanded scope would contribute significantly to our knowledge of carnivore ecology in urbanized landscapes like Kathmandu.

### 4.2 Dietary Niche of Leopard Cats Highlights Its Adaptable Nature

Leopard cats weigh around 4–7 kg, and unlike their larger competitors, exhibit a specialized dietary preference primarily centered around small mammals, especially rodents, as evidenced by diet studies from various regions (Rajaratnam et al. 2007; Shehzad et al. 2012). Our study confirms this specialization, noting that leopard cats in our area exhibit a narrower dietary niche relative to leopards, with rodents being a major dietary component (Table 2). However, it is worth noting their dietary flexibility, as they also consume small birds like passerines and galliformes, fish, and even ungulates to supplement their diet.

Notably, leopard cats pose minimal conflict in urban settings compared to their larger competitors. An average adult leopard cat cannot target medium‐large livestock or pose direct threats to human safety. Instead, given their specialization in a rodent diet, they may indirectly contribute to pest rodent management, particularly in agricultural outskirts, benefiting farming communities (Williams et al. 2018). However, they are often mistakenly accused of poultry predation, leading to unnecessary actions like retaliatory trapping or killings in rural areas (Rai et al. 2018). Our study, through metabarcoding‐based dietary analysis, found no evidence of chicken predation (Table 2), as opposed to its detection in a leopard's diet (Table 1), dispelling misconceptions and promoting informed decision‐making during conflict scenarios related to livestock predation. However, this should be considered with caution given our low sample size, and such decisions need to be made with informed evidence.

Leopard cats may supplement their diet with occasional scavenging of ungulate carrions left by leopards in our study areas. Cattle was detected in one sample from CH, whereas barking deer was found in seven samples, collected from CH, PH, SH, and NJ, because it will be impossible for small felids like leopard cats to bring down juvenile to adult cattle and adult barking deer, with the exception of juvenile barking deer. However, the fawns are usually under the direct protection of their mother doe, making it difficult for them to predate. Such an ecological dynamics emphasizes the role of apex predators as ecosystem engineers in providing meals to small carnivore communities, as outlined by Elbroch et al. (2017) in their study on mountain lions ( *Puma concolor* ) in the USA. Various small carnivore guilds, including felids, mustelids, and viverrids, benefit from such carrions, eventually enhancing overall biodiversity.

We also found leopard cat scats very close to those of leopards, with one scat right above a leopard's scat. Such behavior suggests the interspecies interaction in an ecosystem. Leopard cats, being subordinate to large predators in their habitat, including leopards, may fear scavenging on carrions (Ruprecht et al. 2021) left by the leopards. However, there must be some spatiotemporal separation between these carnivores (Can et al. 2020), which helps the subordinate ones avoid confrontation or being killed by their larger competitors. Such interspecies behavior will also be interesting to study in future research, providing more information on the co‐occurrence of the carnivores in forests affected by urbanization and habitat fragmentation.

## 5 Concluding Remarks

Our study highlights leopard's feeding ecology impacting humans and livestock in the Kathmandu Valley, where forests intersect with human‐use areas. This situation underscores leopards' increasing reliance on domestic animals, creating conflict‐prone hotspots. Mitigating these conflicts is crucial to safeguard human lives and their livestock. Additionally, leopards and other carnivores can offer public health and economic benefits, such as reducing dog bites, lowering sterilization program costs, and decreasing road accidents (Gilbert et al. 2017; Braczkowski et al. 2018). Smaller predators, like leopard cats, play a major role in the food chain involving small mammals like rodents, benefiting local agriculture (Williams et al. 2018). Recognizing these benefits can foster positive attitudes toward carnivores, contributing to their conservation and conflict reduction. However, as leopards prey on domestic animals and indirectly help manage stray populations, it raises concerns about disease transmission between domestic animals and leopards, as these domestic animals bear numerous disease‐causing pathogens and parasites (Thapa, Parajuli, and Dhakal 2022; Adhikari et al. 2023; Adhikari, Dhakal, and Ghimire 2023; Manandhar et al. 2023; Sadaula et al. 2024). This poses health risks to leopards and potentially influences their populations. Habitat disturbance, fragmentation, prey depletion, and increasing encounters with humans due to anthropogenic factors like urbanization may further exacerbate the stress levels of urban leopards, as has been observed in Bengal tigers ( *Panthera tigris* ) in India (Bhattacharjee et al. 2015; Tyagi et al. 2019), potentially affecting their population viability. Future studies should evaluate stress, reproductive physiology, diseases, and genetic health status of leopards to understand the impact of anthropogenic factors on urban leopards. Furthermore, undertaking sex‐id tests to ascertain the sex of the predators that produced the scats and increasing the sample size would contribute to a more comprehensive understanding of the carnivores.

Addressing issues arising from human–wildlife habitat overlaps requires a multifaceted approach. Public education and awareness are critical in fostering tolerance and understanding of the ecological roles of leopards and other carnivores. Promoting the benefits of carnivore presence, such as controlling stray animal populations and reducing public health risks, can help shift public perception (Braczkowski et al. 2018). Additionally, policy interventions and urban planning must prioritize wildlife conservation by creating green corridors and buffer zones to reduce the effects of habitat fragmentation on wildlife. In conclusion, our research provides a baseline overview of the challenges faced by leopards in urbanized regions like the Kathmandu Valley. By addressing habitat fragmentation, reducing conflicts, and fostering positive public attitudes, we can work towards sustainable coexistence with leopards and other carnivores. Ensuring the conservation of leopards will support broader biodiversity goals, benefiting various species within their ecosystems. Effective urban planning and informed wildlife management decisions are essential to achieve these objectives, ultimately securing a harmonious future for both humans and wildlife.

## Author Contributions

**Prajwol Manandhar:** conceptualization (lead), data curation (equal), formal analysis (equal), funding acquisition (lead), methodology (equal), project administration (supporting), software (equal), supervision (equal), visualization (supporting), writing – original draft (lead), writing – review and editing (lead). **Keren S. Pereira:** conceptualization (equal), data curation (equal), formal analysis (equal), methodology (equal), software (equal), visualization (lead), writing – original draft (equal), writing – review and editing (equal). **Naresh Kusi:** conceptualization (supporting), data curation (supporting), investigation (supporting), methodology (supporting), supervision (equal), writing – review and editing (equal). **Jyoti Joshi:** formal analysis (supporting), methodology (equal), resources (supporting). **Noam Levin:** data curation (supporting), formal analysis (supporting), investigation (supporting), methodology (supporting), supervision (supporting), validation (supporting), visualization (supporting), writing – review and editing (equal). **Hemanta K. Chaudhary:** data curation (supporting), formal analysis (supporting), methodology (supporting). **Claudia Wultsch:** investigation (equal), methodology (equal), supervision (equal), validation (equal), writing – review and editing (equal). **Sandesh Lamichhane:** formal analysis (supporting), methodology (supporting), resources (supporting). **Suman Bhandari:** formal analysis (supporting), methodology (supporting), resources (supporting). **Laba Guragain:** methodology (supporting), project administration (supporting), resources (supporting). **Rajesh M. Rajbhandari:** funding acquisition (supporting), investigation (equal), project administration (equal), supervision (supporting). **Berndt J. V. Rensburg:** conceptualization (supporting), investigation (equal), resources (supporting), supervision (supporting), writing – review and editing (equal). **Salit Kark:** conceptualization (supporting), investigation (supporting), supervision (equal), validation (equal), writing – review and editing (supporting). **Dibesh Karmacharya:** conceptualization (supporting), funding acquisition (equal), investigation (supporting), project administration (supporting), resources (equal), supervision (equal), validation (equal), writing – review and editing (lead).

## Conflicts of Interest

The authors declare no conflicts of interest.

## Supporting information


Table S1.
Table S2.
Table S3.
Table S4.

## Data Availability

We have deposited all the *Cytochrome b* sequences generated in this study in the NCBI GenBank database with accession numbers ON364493–ON364521. The Vertebrate prey *12S‐V5* sequence fastq data generated by Illumina MiSeq in the present study were deposited in the NCBI SRA database under BioProject accession number PRJNA1137159 and are accessible via https://www.ncbi.nlm.nih.gov/sra/PRJNA1137159. The data on the percentage occurrence of prey species of each individual scat will be provided upon request to the corresponding author.
