# Supplementary material for: Feeding Habits of Leopards and Leopard Cats in the Fragmented Forests Surrounding the Kathmandu Valley
Source: Ecol Evol. 2025 Jan 29;15(2):e70927. doi: 10.1002/ece3.70927 (PMC11775411; doi:10.1002/ece3.70927)
Supplement: Supplementary file 1 — Table S1. Table S2. Table S3. Table S4. [file ECE3-15-e70927-s001.docx]

**Supplementary Tables**

**Supplementary Table 1.** Primers used in this study. Primers for Leopard species-id were self-designed for this particular research. These primers target the mitochondrial *Cytochrome-b* gene of Leopard *Panthera pardus* and amplifies ~200bp region, which can be visualized via Gel electrophoresis.

| **Purpose** | **Primers name** | **Sequence (5' to 3')** | **Product size** | **Reference** |
| --- | --- | --- | --- | --- |
| Leopard species id | Ppa-Sf | CCCCGCTCCATCCAACATCTCAA | ~200bp | This study* |
|  | Ppa-Sr | GGCTCCATTGGCGTGTAGATAC |  |  |
| Universal DNA barcoding | mcb398 | TACCATGAGGACAAATATCATTCTG | ~412bp | Verma & Singh 2004 |
|  | mcb869 | TCCTAGTTTGTTAGGGATTGATCG |  |  |
| Vertebrate prey metabarcoding | 12SV5F | TTAGATACCCCACTATGC | ~100bp | Riaz et al., 2011 |
|  | 12SV5R | TAGAACAGGCTCCTCTAG |  |  |
| Leopard 12S blocking | PantB | CTATGCTTAGCCCTAAACCTAGATA-GTTAGCCCAAACAAAACTAT | 3’ C3 spacer | Shehzad et al., 2014 |

*Annealing temperature of the leopard specific primers is 60°C

**Supplementary Table 2.** FRAGSTATS class metrics (see Southworth et al. 2004; Miyamoto and Sano, 2008; Midha and Mathur, 2010).

| **Metric** | **Unit** | **Description** | **Range** | **Fragmentation predictors** |
| --- | --- | --- | --- | --- |
| Patch Density (PD) | patches/100ha | Number of patches of the corresponding *class divided by total **landscape area, multiplied by 100.  Greater density of patches of corresponding class will indicate it has been broken into many patches thus, could be considered more fragmented. | PD > 0, constrained by cell size | PD ↑ = Fragmentation likelihood ↑ |
| Edge Density (ED) | m/ha | Total length of edge of the corresponding class divided by total area.  Amount of edge length relative to total class area in the landscape is expected to increase if there is fragmentation. | ED ≥ 0, without limit | ED ↑ = Fragmentation likelihood ↑ |
| Mean Patch Area (MPA) | Ha | Patch area divided by 10,000.  Smaller MPA is expected to indicate the corresponding class is fragmented. | AREA > 0, without limit | MPA ↓ = Fragmentation likelihood ↑ |
| Mean Shape Index (MSI) | - | Average shape index of patches of corresponding class, adjusted by a constant for a square standard (raster).  Patches that are more geometrically complex indicate the corresponding class is more fragmented. | SHAPE ≥ 1, without limit | MSI ↑ = Fragmentation likelihood ↑ |
| Mean Core Area (MCA) | ha | Average core area of the patches of the corresponding class.  Amount of core area will decrease if there is fragmentation as more core habitat is converted into edge habitat. | CORE ≥ 0, without limit | MCA ↓ = Fragmentation likelihood ↑ |
| Mean Euclidean Nearest-Neighbour Distance (MNN) | m | Average distance between a class’ patch and its nearest neighbour, based on edge to edge distance.  As patches become increasingly smaller and more isolated this distance is expected to increase. | ENN > 0, without limit | ENN ↑ = Fragmentation likelihood ↑ |

**Supplementary Table 3.** FRAGSTATS fragmentation metric values for forests in the entire study area and the six major surveyed forest patches. The class metrics read as total class area (CA), percentage of landscape (PLAND), patch density (PD), edge density (ED), mean patch area (MPA), mean shape index (MSI), mean core area (MCA) and mean Euclidean nearest neighbour distance (MNN).

| **Forest** | **CA (ha)** | **PLAND (%)** | **PD (patches/100 ha)** | **ED (m/ha)** | **MPA (ha)** | **MSI** | **MCA (ha)** | **MNN (m)** |
| --- | --- | --- | --- | --- | --- | --- | --- | --- |
| Study area | 42895.1 | 46.0 | 3.4 | 43.1 | 13.7 | 1.3 | 8.3 | 98.7 |
| Shivapuri | 11800.9 | 75.9 | 2.1 | 38.3 | 36.6 | 1.3 | 27.0 | 87.6 |
| Nagarjun | 1916.6 | 83.8 | 1.4 | 25.7 | 61.8 | 1.2 | 50.1 | 89.6 |
| Indradaha | 865.5 | 55.1 | 4.1 | 86.5 | 13.5 | 1.5 | 4.5 | 74.7 |
| Chandragiri | 6919.8 | 78.5 | 1.4 | 34.4 | 54.9 | 1.2 | 40.3 | 81.6 |
| Phulchowki | 9443.3 | 80.9 | 1.8 | 42.4 | 45.4 | 1.3 | 32.6 | 77.5 |
| Nagarkot | 1203.5 | 85.3 | 2.1 | 45.7 | 41.5 | 1.3 | 27.3 | 73.8 |

**Supplementary Table 4.** Results of the two-way ANOVAs with the three explanatory variables that explain the effect of habitat disturbance on the diets of the two carnivore species.

| **Explanatory variables** | **Two-way ANOVA output** |
| --- | --- |
| Proportion of forest 1000 m around the scats | F=1.49, N=46, df=1, *p=0.22* |
| Distance of scats from the nearest forest edge | F=0.23, N=46, df=1, *p=0.63* |
| Level of forest fragmentation 1000 m around the scats | F=0.21, N=46, df=1, *p=0.65* |
